# Fueling thermogenesis: an individual-level thermography assay reveals that sucrose ingestion promotes thermogenesis in honeybees

**DOI:** 10.1242/jeb.252296

**Published:** 2026-09-14

**Authors:** Saskia Delac, Christine von Koschitzky, Markus Thamm

**Affiliations:** Department of Behavioral Physiology and Sociobiology, University of Würzburg, 97070 Würzburg, Germany

**Keywords:** *Apis mellifera*, Carbohydrate, Crop content

## Abstract

Western honeybees (*Apis mellifera*) are remarkable among insects for their ability to generate heat endogenously and maintain a stable hive microclimate. This process relies on the activity of the indirect flight muscles, but the direct relationship between thermogenesis and carbohydrate availability has not been systematically investigated. To investigate this relationship, we developed a controlled individual-level thermography assay that provides a standardized framework for studying honeybee thermogenesis at the individual level. Starved bees showed little or no thermogenesis, whereas sucrose-fed bees exhibited substantially increased thoracic temperatures and thermogenic activity, consistent with a threshold-like activation of thermogenesis. Additionally, thermogenic performance was significantly lower in bees fed a sucralose solution used as a putatively non-nutritive control than in bees that received the same volume of sucrose solution. Together, these findings support the hypothesis that thermogenesis depends on the availability of recently ingested carbohydrates.

## Teaser

**Summary:** A new individual-level thermography assay shows that thermogenesis in honeybees depends on sucrose ingestion in a threshold-like manner, regardless of whether bees are nurses or foragers.

## INTRODUCTION

Most insects lack the ability to actively maintain their body temperature over an extended period of time (Lahondère, 2023). The western honeybee (*Apis mellifera*), however, is notable among insects because of its eusocial traits, which enable thermoregulatory strategies. These include cooling (evaporation of water and fanning) and heating (shivering thermogenesis) (Lindauer, 1955; Kronenberg and Heller, 1982), making colonies relatively independent of environmental fluctuations and ensuring thermal homeostasis within a beehive (Stabentheiner et al., 2021; Straub et al., 2015) over extended periods of time. Thermoregulation is essential to ensure optimal brood temperatures and the long-term survival of the colony. Significant temperature deviations from the normal state (∼35°C; Himmer, 1932) have the potential to result in developmental abnormalities (Himmer, 1932), alter neural organization in the adult bee brain (Groh et al., 2004), affect behavioral performance (Tautz et al., 2003) and, ultimately, increase mortality rates. Shivering thermogenesis especially is a remarkable ability that allows honeybees to actively increase their body temperature (Kovac et al., 2009; Stabentheiner et al., 2010). This is made possible by the indirect flight muscles, located in the thorax of a honeybee (Markl, 1966). These muscles primarily consist of the dorsoventral muscles and the large dorsolongitudinal muscle. The flight muscles produce a smooth tetanus through the summation of rapid neurogenic contractions which result in the generation of heat (Esch et al., 1991; Esch and Goller, 1991). This active heat production is often carried out by nurse bees and contributes not only to maintaining thermal homeostasis within a hive but also to defense against predatory hornets (Ken et al., 2005; Baracchi et al., 2010), winter survival of a colony (Stabentheiner et al., 2003) and the pre-flight warm-up of the muscles (Esch and Goller, 1991). Although flight muscles are central to thermogenesis, their primary function is to power flight for foraging trips performed by forager bees. The flight metabolism of honeybees is better understood than their thermogenic mechanisms. Flight is among the most energy-demanding biological processes known, with honeybee flight muscles exhibiting some of the highest metabolic rates and power outputs recorded in the animal kingdom (Suarez et al., 1996; Feuerbacher et al., 2003; Hickey et al., 2022; Suarez, 2000). Consequently, sufficient energy reserves are required for honeybees to power the exclusively aerobic metabolic pathways for flight (Rothe and Nachtigall, 1989; Suarez et al., 1996). These pathways primarily rely on hexose sugars such as glucose and fructose (Bounias, 1981; Arslan et al., 1986), rather than on energy stores such as glycogen (Panzenböck and Crailsheim, 1997). Flight requires up to 1.14 μl O_2_ g^−1^ min^−1^ per action potential, where each action potential leads to around ten muscle contractions (Bastian and Esch, 1970; Heinrich, 2013). Interestingly, thermogenesis exhibits comparable metabolic demands, with oxygen consumption rates of about 1.16 μl O_2_ g^−1^ min^−1^ per action potential. However, during thermogenesis, action potentials and muscle contractions occur at an approximately 1:1 ratio (Bastian and Esch, 1970; Heinrich, 2013). Although flight and thermogenesis are both facilitated by the indirect flight muscles, with similar metabolic rates, the direct role of carbohydrate intake in sustaining thermogenesis remains largely unexplored. In a previous study, Kaya-Zeeb et al. (2022) found evidence suggesting that octopaminergic signaling activates glycolysis via protein kinase A pathways during thermogenesis. While this does not provide a direct link between thermogenesis and carbohydrate intake, it suggests that thermogenesis requires carbohydrate metabolism. Additionally, Heinrich stated that ‘Honeybees generally remain endothermic as long as they have sugar in their honey-stomach’ (Heinrich, 2013).

However, whether thermogenesis directly depends on available carbohydrate intake has not yet been systematically investigated under laboratory conditions. Moreover, to our knowledge, no standardized individual-level assay is currently available for the controlled quantification of thermogenic performance in honeybees. Therefore, in the present study, we quantified thermogenic responses in nurse and forager bees following ingestion of defined sucrose solutions. The study aimed to assess whether the ingestion of sucrose solution is associated with inducing thermogenesis, based on the premise that flight muscle contractions depend on accessible carbohydrate energy sources (Suarez et al., 1996). Given that nurse bees primarily engage in colony thermogenesis, whereas forager bees rely on flight muscle activity for flight – with both activities having comparable metabolic demands (Bastian and Esch, 1970; Heinrich, 2013) – we included both worker bee groups in independent experiments to assess whether the effects of sucrose ingestion on thermogenesis could be observed across different behavioral contexts.

## MATERIALS AND METHODS

### Animals

Honeybees (*Apis mellifera carnica* Pollman 1879) were collected from departmental colonies (Biocenter, University of Würzburg, Germany). Nurse and forager bees were classified as described in Kaya-Zeeb et al. (2022) and collected at different time points based on their differences in task and age. The gathered animals were caged, placed in an incubator (30°C, 50% RH, dark conditions) and fed with 30% (w/v) sucrose solution *ad libitum* overnight.

### Thermography based on food quantity

**Fig. 1.**
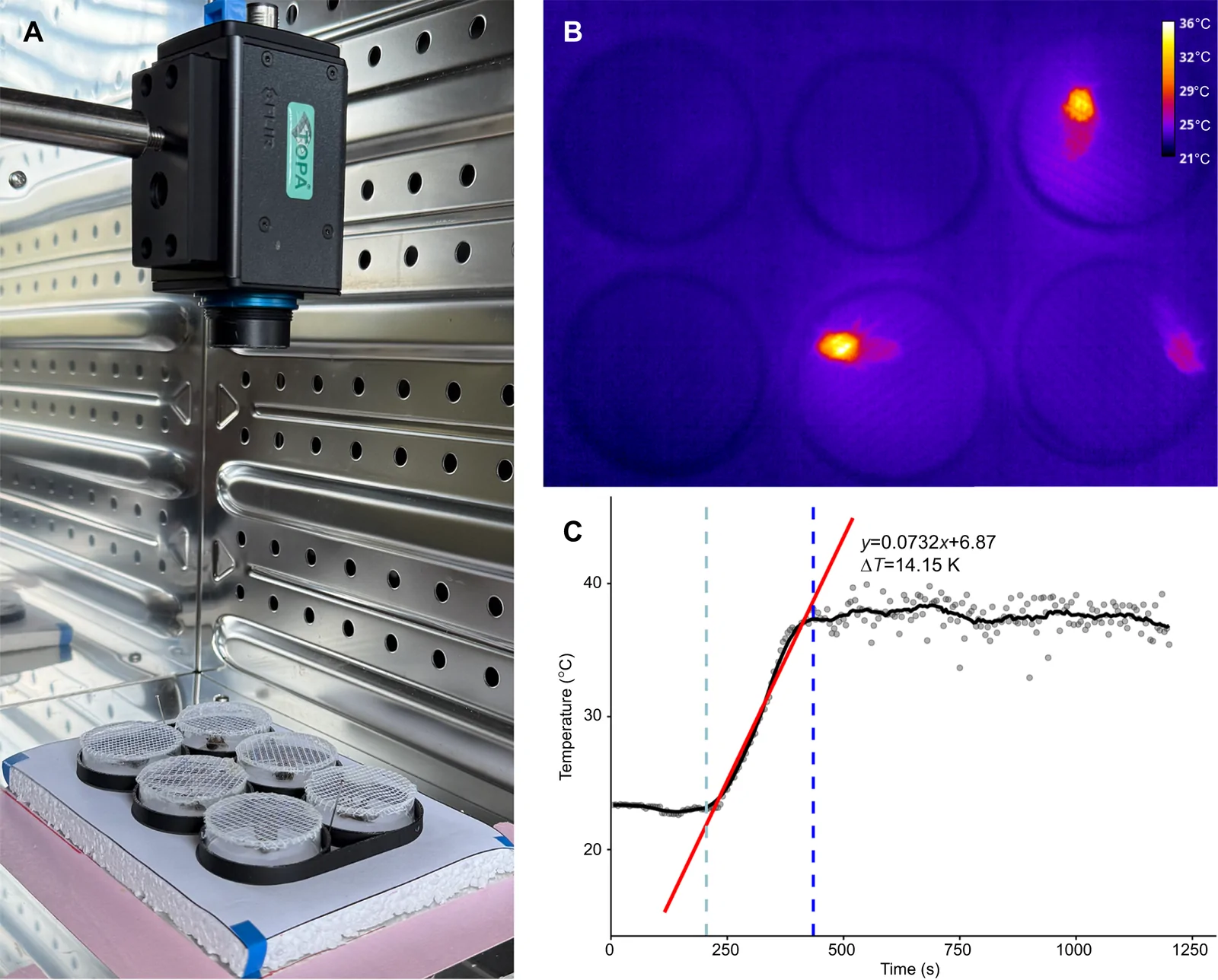
**Thermographic setup to assess thermogenesis in honeybees.** (A) Our setup includes a thermal imaging camera and a plate made from expanded polystyrene with immobilized bees beneath modified Petri dishes. (B) A thermographic image captured during the experiment shows individual bees performing thermogenesis. While some bees exhibit significant thermal activity, demonstrating their ability to generate heat, others show little to no thermogenic response. (C) An example evaluation of the heating capacity of a single heating bee. Grey dots represent raw temperature data and the black line the smoothed temperature data. A heating event has a classified start (dotted light blue line) and endpoint (dotted dark blue line). In between, a linear regression (*y*=0.0732*x*+6.87) was fitted (red line) and the temperature difference (14.15 K) calculated.

Thermogenesis performance of nurse and forager bees was analyzed in relation to food quantity. Food was withdrawn 4 h prior to measurements, as previous tests with forager bees had shown that honey stomachs are empty after this period of starvation (Fig. S1). Following the fasting interval, bees were fed either 0 μl, 10 μl or 20 μl of 30% (w/v) sucrose solution (volume experiment), immobilized on ice and placed on an expanded polystyrene plate. To assess whether thermogenesis depends on caloric availability rather than crop volume alone, a sucralose versus sucrose experiment was conducted. Sucralose (Sigma-Aldrich) was chosen as a putative non-metabolizable sucrose analogue based on Price et al. (2023) showing that it is non-nutritive in *Vespula pensylvanica* and *Drosophila suzukii*, suggesting that honeybees may similarly be unable to utilize sucralose as an energy source. To obtain a preliminary indication of the ability of sucralose alone to sustain survival in *A. mellifera*, an exploratory assay was conducted (Fig. S2). Sucralose was prepared at 1% (w/v), the highest concentration accepted during preliminary tests. For this experiment, bees were immobilized on ice to individually restrain them in proboscis extension response (PER) holders (Bitterman et al., 1983) at 2 h and 45 min into the fasting interval and returned to the dark incubator (30°C) for the remaining starvation period. At 3 h and 30 min, bees were removed from the incubator and fed individually by presenting either 1% (w/v) sucralose solution or 30% (w/v) sucrose solution to the antennae to elicit proboscis extension, after which bees were allowed to consume 20 μl of the respective solution. This earlier endpoint of starvation was necessary to accommodate the larger number of animals tested (Table S1). Bees that accepted 20 μl sucralose or sucrose solution were subsequently immobilized on ice, taken out of their PER holders and placed on the polystyrene plate as described above. Each bee was then covered with a modified Petri dish (3 cm diameter) with the bottom replaced with a fine mesh net oriented upward to allow air exchange and enable video recording with view of the thorax. The setup was transferred into an incubator (18°C, dark conditions) and measurements were started 2 min after placement. The incubator was equipped with a thermal imaging camera (FLIR A65 camera, lens: 45 deg, *f*=13 mm, FLIR, Wilsonville, USA; Fig. 1A). At an emissivity of 0.97 (Stabentheiner and Schmaranzer, 1987), thermal images of each bee were recorded at 30 frames s^−1^ for 20 min at 5 s intervals using the FLIRTools+ program (Fig. 1B). Thermographic videos were converted from FLIR radiometric .seq format to calibrated temperature matrices using the open-source R package Thermimage (v.4.1.3; https://github.com/gtatters/Thermimage). Following this, the thoracic temperatures of honeybees were read with ImageJ (v.1.53 c; Schindelin et al., 2012). A circular region of interest (ROI) matching the diameter of the Petri dish was defined, encompassing a honeybees full movement area. Within this ROI, the maximum pixel temperature was extracted over time, corresponding to the thorax as the warmest region, thereby avoiding spatial averaging with the mesh. The obtained temperature data was smoothed using the R package zoo (v. 1.8.14; https://CRAN.R-project.org/package=zoo; Zeileis and Grothendieck, 2005) and analyzed for characteristic heating phases. Phases were identified using defined threshold values, and a linear regression was fitted to each heating phase (Fig. 1C). In order to differentiate heating from non-heating individuals, we applied three criteria. An individual could only be classified as a ‘heater’ if all three of the following criteria were met: (1) a distinct onset of a heating phase occurred, (2) the slope of the linear regression was positive and (3) the final thorax temperature exceeded 30°C. A detailed step-by-step description of the ImageJ and R analysis workflow is provided in figshare (doi:10.6084/m9.figshare.32778639).

### Statistical analysis

All further statistical analyses were performed using R (v.4.6.0; r-project.org), RStudio (v.2026.01.0) and the R packages rstatix (0.7.3; https://CRAN.R-project.org/package=rstatix) and rcompanion (v.2.5.2; https://CRAN.R-project.org/package=rcompanion). Data preparation was performed using dplyr (v.1.2.1; https://CRAN.R-project.org/package=dplyr), tidyr (v.1.3.1; https://CRAN.R-project.org/package=tidyr), purrr (v.1.2.2; https://CRAN.R-project.org/package=purrr), tibble (v.3.3.1; https://CRAN.R-project.org/package=tibble), reshape2 (v.1.4.5; https://CRAN.R-project.org/package=reshape2) and stringr (v.1.6.0; https://CRAN.R-project.org/package=stringr). A Shapiro–Wilk test was performed to assess the data for normal distribution. As most of the data subsets did not display a normal distribution, the data was further analyzed using the Kruskal–Wallis test, followed by Dunns *post hoc* analysis with Holm correction. The proportion of heaters and non-heaters across treatment groups was compared using a Fisher's exact test with Bonferroni correction for pairwise comparisons. Since only two groups were compared in the sucralose experiment, a Wilcoxon rank-sum test was applied (see Table 1). Data visualisation was carried out using the R packages ggplot2 (v.4.0.3; https://CRAN.R-project.org/package=ggplot), ggpubr (v.0.6.3; https://CRAN.R-project.org/package=ggpubr), gghalves (v.0.1.4; https://CRAN.R-project.org/package=gghalves), ggnewscale (v.0.5.2; https://CRAN.R-project.org/package=ggnewscale) and scales (v.1.4.0; https://CRAN.R-project.org/package=scales). Inkscape (v.1.4.2) was used to arrange images.

**Table 1.** Statistical analysis of the thermogenesis performance data

	Analysis	Test	Groups (*n*)	Results
Nurse bees				
Volume experiments	Slope (°C s^−1^)	Kruskal–Wallis test		χ^2^=12.3, d.f.=2, **P=0.0021
		Dunns test (Holm)	0 μl (15) vs 10 μl (15)	*Z*=0.806, *P*_adj_=1.00 (n.s.)
			0 μl (15) vs 20 μl (15)	*Z*=3.36, **P_adj_=0.0023
			10 μl (15) vs 20 μl (15)	*Z*=2.56, *P_adj_=0.0316
	*T*_max_ (°C)	Kruskal–Wallis test		χ^2^=13.7, d.f.=2, **P=0.0011
		Dunns test (Holm)	0 μl (15) vs 10 μl (15)	*Z*=2.11, *P*_adj_=0.0692 (n.s.)
			0 μl (15) vs 20 μl (15)	*Z*=3.68, ***P_adj_=0.0007
			10 μl (15) vs 20 μl (15)	*Z*=1.57, *P*_adj_=0.116 (n.s.)
	Non-heater versus heater	Fisher's exact test		**P=0.010
		Pairwise Fisher's exact test (Bonferroni)	0 μl (15) vs 10 μl (15)	*P*_adj_=1.00 (n.s.)
			0 μl (15) vs 20 μl (15)	*P_adj_=0.0233
			10 μl (15) vs 20 μl (15)	*P*_adj_=0.1970 (n.s.)
		
Sucralose experiments	Slope (°C s^−1^)	Wilcoxon rank-sum test	Sucralose (17) vs sucrose (18)	*W*=35, ****P=0.00003
	*T*_max_ (°C)	Wilcoxon rank-sum test	Sucralose (17) vs sucrose (18)	*W*=39, ****P=0.00007
	Non-heater versus heater	Fisher's exact test	Sucralose (17) vs sucrose (18)	****P=0.00008
Forager bees				
Volume experiments	Slope (°C s^−1^)	Kruskal–Wallis test		χ^2^=14.3, d.f.=2, ***P=0.0008
		Dunns test (Holm)	0 μl (17) vs 10 μl (19)	*Z*=2.11, *P*_adj_=0.0691 (n.s.)
			0 μl (17) vs 20 μl (19)	*Z*=3.78, ***P_adj_=0.0005
			10 μl (19) vs 20 μl (19)	*Z*=1.71, *P*_adj_=0.0870 (n.s.)
	*T*_max_ (°C)	Kruskal–Wallis test		χ^2^=18.1, d.f.=2, ***P=0.0001
		Dunns test (Holm)	0 μl (17) vs 10 μl (19)	*Z*=2.89, **P_adj_=0.0076
			0 μl (17) vs 20 μl (19)	*Z*=4.17, ****P_adj_=0.00009
			10 μl (19) vs 20 μl (19)	*Z*=1.32, *P*_adj_=0.1880 (n.s.)
	Non-heater versus heater	Fisher's exact test		****P=0.00004
		Pairwise Fisher's exact test (Bonferroni)	0 μl (17) vs 10 μl (19)	**P_adj_=0.0038
			0 μl (17) vs 20 μl (19)	****P_adj_=0.00003
			10 μl (19) vs 20 μl (19)	*P*_adj_=0.885 (n.s.)
		
Sucralose experiments	Slope (°C s^−1^)	Wilcoxon rank-sum test	Sucralose (17) vs sucrose (21)	*W*=33, ****P=0.000004
	*T*_max_ (°C)	Wilcoxon rank-sum test	Sucralose (17) vs sucrose (21)	*W*=28, ****P=0.000001
	Non-heater versus heater	Fisher's exact test	Sucralose (17) vs sucrose (21)	***P=0.0002

*P_adj_<0.05, **P_adj_<0.01, ***P_adj_<0.001, ****P_adj_<0.00001, n.s., not significant (*P*_adj_≥0.05).

## RESULTS AND DISCUSSION

**Fig. 2.**
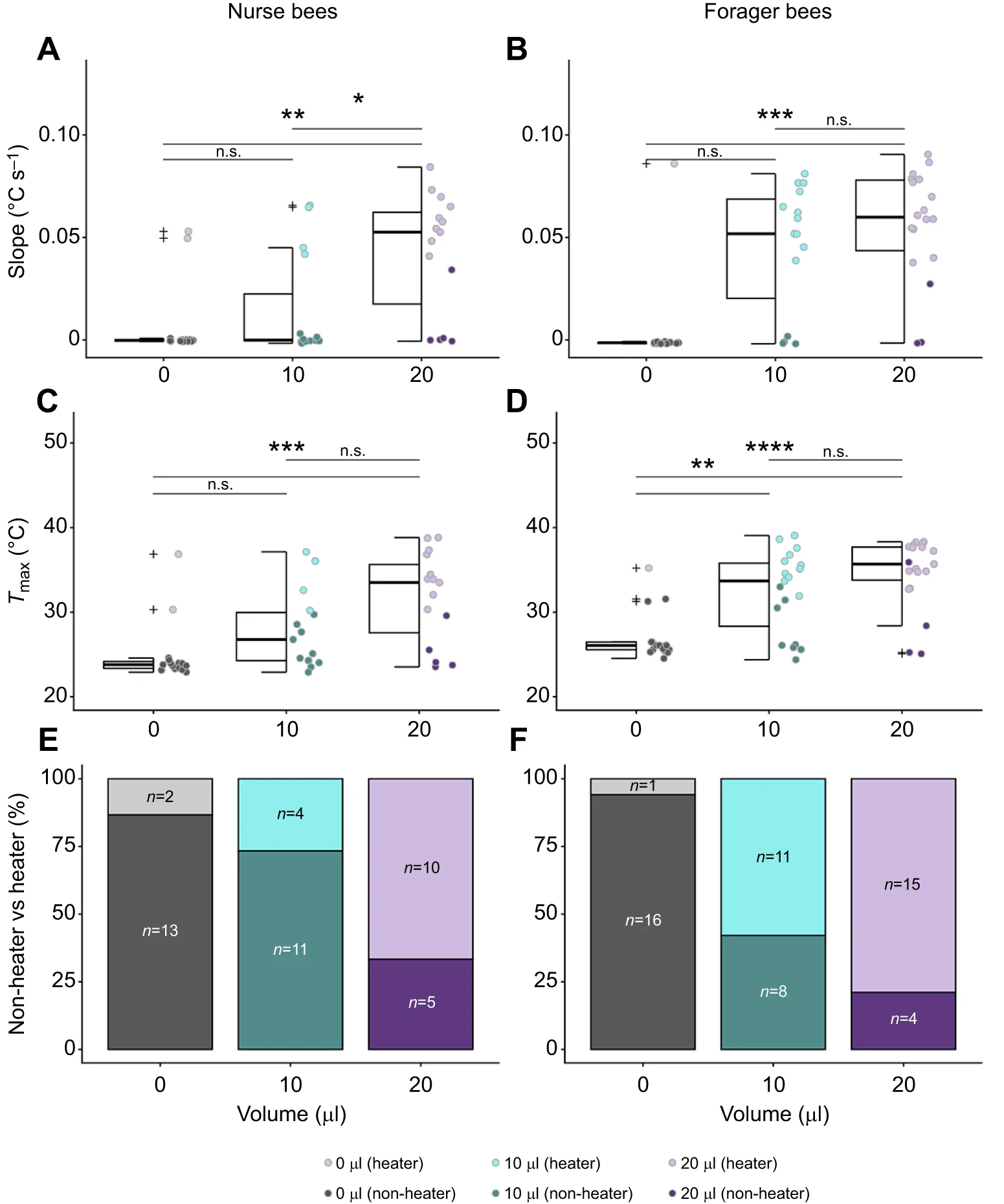
**Effects of feeding volume on thermogenesis in honeybees.** (A–F) Varying volume experiment in nurse bees (A,C,E) and forager bees (B,D,F). (A,B) The rate of temperature change (slope) and (C,D) the maximum thoracic temperature. Boxes show the 25–75th percentiles with median; whiskers show the 1.5× interquartile range along with individual data points on the right. Data points are color-coded according to consumed food volume and heater classification. Outliers are represented as crosses. *P_adj_<0.05, **P_adj_<0.01, ***P_adj_<0.001, ****P_adj_<0.00001, n.s., not significant (*P*_adj_≥0.05). (E,F) The proportion of honeybees classified as non-heater or heater. Darker colors indicate non-heaters, lighter colors indicate heaters. For detailed statistics and sample sizes, see Table 1.

**Fig. 3.**
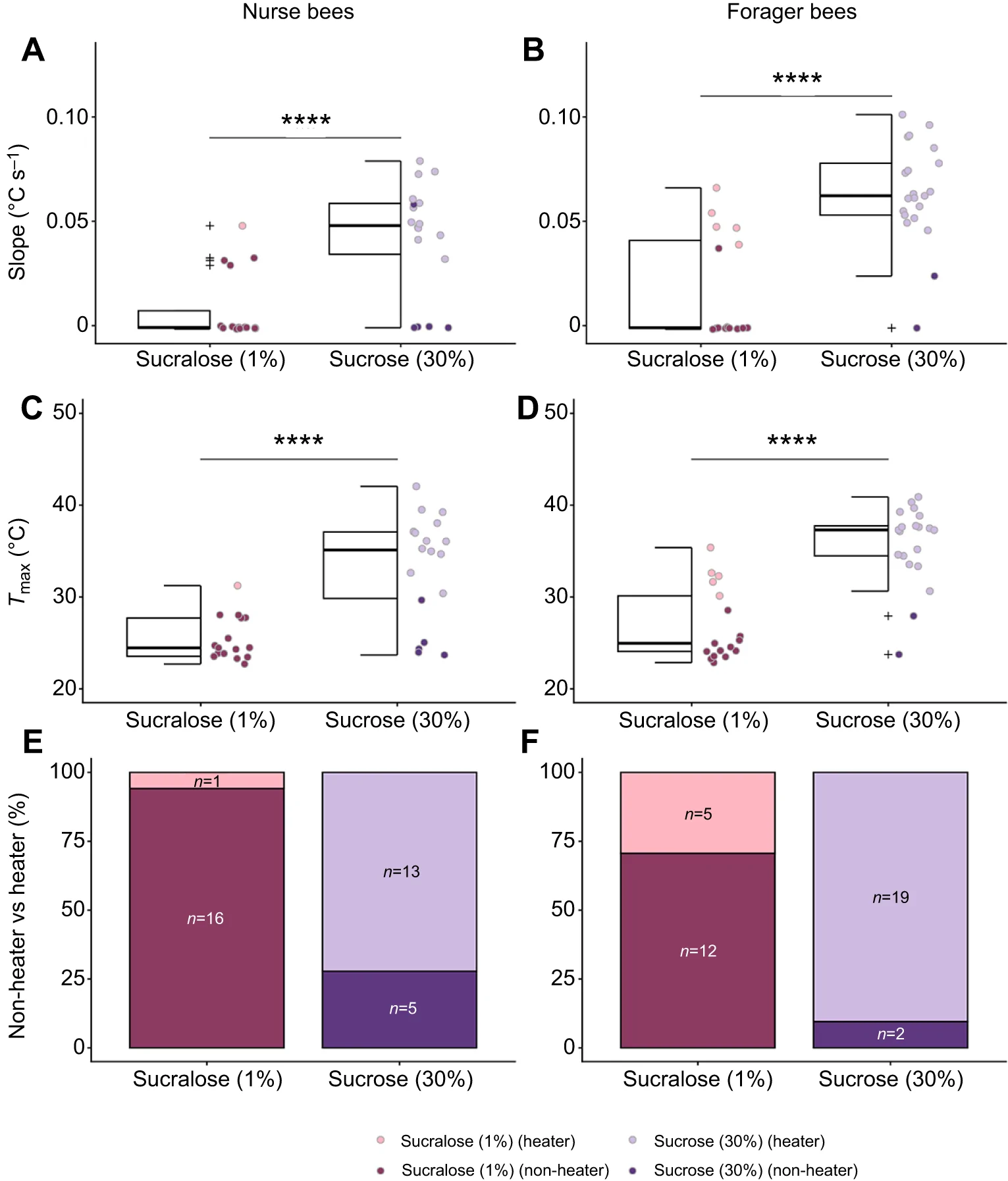
**Effects of ingested substance on thermogenesis in honeybees.** (A–F) 1% sucralose versus 30% sucrose experiment at a fixed volume of 20 μl in nurse bees (A,C,E) and forager bees (B,D,F). (A,B) The rate of temperature change (slope) and (C,D) the maximum thoracic temperature. Boxes show the 25–75th percentiles with median; whiskers show the 1.5× interquartile range along with individual data points on the right. Data points are color-coded according to consumed substance and heater classification. Outliers are represented as crosses. ****P_adj_<0.00001. (E,F) The proportion of honeybees classified as non-heater or heater. Darker colors indicate non-heaters, lighter colors indicate heaters. For detailed statistics and sample sizes, see Table 1.

In order to systematically investigate the effect of carbohydrate availability on thermogenesis behavior, honeybees were fed different volumes of 30% sucrose solution. Honeybees that were not allowed to feed (0 μl) hardly exhibited any thermogenesis. Their rate of temperature change was low (Fig. 2A,B, Table 1) and their maximum thoracic temperature was the lowest among all groups (Fig. 2C,D, Table 1). Furthermore, non-heaters were most prevalent (Fig. 2E,F, Table 1). This probably reflects a decline in hemolymph sugar levels (Abou-Seif et al., 1993), including glucose and fructose (Maurizio, 1965; Barker and Lehner, 1974; Bounias, 1981; Arslan et al., 1986), combined with the inherently low glycogen reserves of honeybees (Neukirch, 1982; Panzenböck and Crailsheim, 1997) after starvation. Furthermore, it seems that the remaining carbohydrate substrates are insufficient to sustain enough ATP generation for thermogenic muscle activity (Fig. 2A–F, Table 1). Honeybees fed with 20 μl of 1% sucralose solution showed low rates of temperature increase (Fig. 3A,B, Table 1) and low maximum thoracic temperature (Fig. 3C,D, Table 1), with the majority being classified as non-heaters (Fig. 3E,F, Table 1), resembling the pattern observed in starved bees (0 μl sucrose; Fig. 2A–F, Table 1). The reduced thermogenic performance suggests that putatively non-nutritive sugar analogues are insufficient to facilitate thermogenesis in *Apis mellifera*. Since sucralose-fed bees received the same volume as the highest sucrose treatment (20 μl), yet failed to exhibit thermogenesis, a purely volumetric signal, such as crop stretch, is unlikely to account for thermogenesis. However, it should be noted that sucralose was used at a lower concentration than sucrose (1% versus 30%) owing to the honeybees’ resistance to ingest higher concentrations. Sucralose was selected to provide an equivalent volume without offering metabolically accessible carbohydrates, as studies have shown this compound to be non-nutritive in other hymenopteran and dipteran species (Price et al., 2023). To test this for *Apis mellifera*, the survival of sucralose-fed and sucrose-fed bees was compared in an exploratory assay. All bees provided with 1% sucralose died within the observation period (median survival=330 min), whereas most bees provided with 30% sucrose survived until the end of the observation period, resulting in a significant difference in survival between treatments (Fig. S2). These results are consistent with the idea that sucralose alone is not enough to ensure survival, though the comparison was limited to these two feeding conditions. Moreover, the low acceptance rate of the sucralose solution probably indicates gustatory aversion (Table S1). Consequently, the sucralose group may represent a subset that is biased in terms of motivation, which should be considered when interpreting the comparison with sucrose-fed bees. Therefore, the results should be interpreted as being consistent with a role of carbohydrate availability in thermogenesis, while acknowledging that the experimental design does not fully exclude alternative explanations related to treatment acceptance or motivational differences between groups.

In contrast, honeybees provided with sucrose solution exhibited thermogenesis in a threshold-like manner. The most pronounced difference was observed between starved and fed bees, whereas thermogenesis performance differed less considerably between intermediate (10 μl) and high feeding volumes (20 μl, Fig. 2A–D, Table 1). This pattern is consistent with a threshold-like rather than a graded dose-response relationship. Nurse and forager bees displayed the most evident signs of thermogenesis only after 20 μl had been provided. Collectively, these findings indicate that thermogenesis in honeybees requires metabolically accessible carbohydrates, consistent with the dependence of flight muscle ATP generation on circulating sugars rather than on volumetric or gustatory signals alone. The threshold-like dependence of thermogenesis on carbohydrate availability is consistent with the rapid intestinal hydrolysis of sucrose into glucose and fructose (Kosmin and Komarow, 1932) and their fast absorption into the hemolymph (Maurizio, 1965; Crailsheim, 1988b). These hexose sugars may subsequently be transported to the flight muscles (Barker and Lehner, 1974), where they can support the energy-demanding activity of the flight muscles and thereby contribute to heat production. Given the extremely high energetic demands of flight, up to 10% of a honeybee's body mass in carbohydrates per hour (Dietz, 1975) and the limited ATP yield from minimal glycogen reserves (Crailsheim, 1988a; Panzenböck and Crailsheim, 1997; Southwick, 1991), it is unlikely that flight muscles can rely solely on internal energy reserves.

Once sucrose was provided, both nurse and forager bees showed improved thermogenic performance (Fig. 2A,B, Table 1), greater maximum thoracic temperature (Fig. 2C,D, Table 1) and the highest proportion of heating individuals (Fig. 2E,F, Table 1). These observations are consistent with the hypothesis that sufficient carbohydrate availability is a prerequisite for thermogenesis in both worker bee groups. The shared carbohydrate dependency of thermogenesis across worker groups may contribute to the metabolic flexibility that underlies task reallocation in the colony, although this remains to be directly tested. Carbohydrate intake generally promotes thermogenesis, but it did not fully account for individual variation in thermogenesis response within the feeding groups. While most individuals performed thermogenesis after ingesting 20 μl sucrose solution, a subset of bees was classified as non-heaters. This included four nurse bees and five forager bees in the volume experiment, as well as five nurse bees and two forager bees in the sucrose control group of the sucralose experiment (Fig. 2E,F, Fig. 3E,F). These observations suggest that thermogenesis depends on additional factors, such as prior experience and age (Stabentheiner et al., 2010). Age-related differences may reflect not only behavioral specialization but also the maturation of flight muscles and associated changes in metabolic enzyme composition, which are known to influence endothermic and flight capacity (Hersch et al., 1978; Harrison, 1986; Moritz, 1988; Menail et al., 2023). Moreover, it is important to consider that thermogenesis in honeybees often involves a social component. For instance, heating occurs in the presence of brood (Koeniger, 1978; Bujok et al., 2002; Kleinhenz et al., 2003; Stabentheiner et al., 2010), to supply and protect the colony (Esch and Goller, 1991; Ken et al., 2005; Baracchi et al., 2010) or to ensure their winter survival (Stabentheiner et al., 2003). In our experimental setup, such social cues were intentionally absent to allow precise control of individual behavior and physiology. This ‘sterile’ environment may, however, reduce the motivation of some individuals to engage in thermogenesis. Future studies should aim to identify which social or environmental factors promote thermogenesis and, where possible, incorporate them into controlled laboratory experiments.

Taken together, our results emphasize that a constant supply of carbohydrates is essential, indicating that even a starvation period of four hours is sufficient to substantially impair thermogenic capacity. Given this sensitivity to carbohydrate intake, these findings suggest that thermogenesis depends on the resources available within a colony. Accordingly, sufficient carbohydrate reserves (honey) are probably critical during brood rearing and before winter. However, maintaining sufficient reserves is not always possible for natural and managed colonies. Natural colonies may experience seasonal food scarcity due to limited or fluctuating floral resources (Kohl et al., 2023). In managed colonies, several factors such as excessive honey removal, poor supplement feeding (Islam et al., 2025) and periods of resource limitation (Lowe et al., 2023) may lead to food shortages. Consequently, these shortages have the potential to compromise thermogenesis, thereby exerting a negative influence on brood development and colony growth (Himmer, 1932; Tautz et al., 2003; Groh et al., 2004).

Collectively, our results support the hypothesis that thermogenesis in honeybees depends on the availability of recently ingested carbohydrates, independent of worker task. Beyond the biological findings presented here, the controlled individual-level assay provides a foundation for future studies to investigate the physiology, pharmacology and nutritional regulation of thermogenesis in honeybees.

## Supplementary Material

10.1242/jexbio.252296_sup1 Supplementary information
