# Supplementary material for: Fueling thermogenesis: an individual-level thermography assay reveals that sucrose ingestion promotes thermogenesis in honeybees
Source: J Exp Biol. 2026 Sep 14;229(18):jeb252296. doi: 10.1242/jeb.252296 (PMC13615678; doi:10.1242/jeb.252296)
Supplement: Supplementary file 1 [file jexbio-229-252296-s1.pdf]

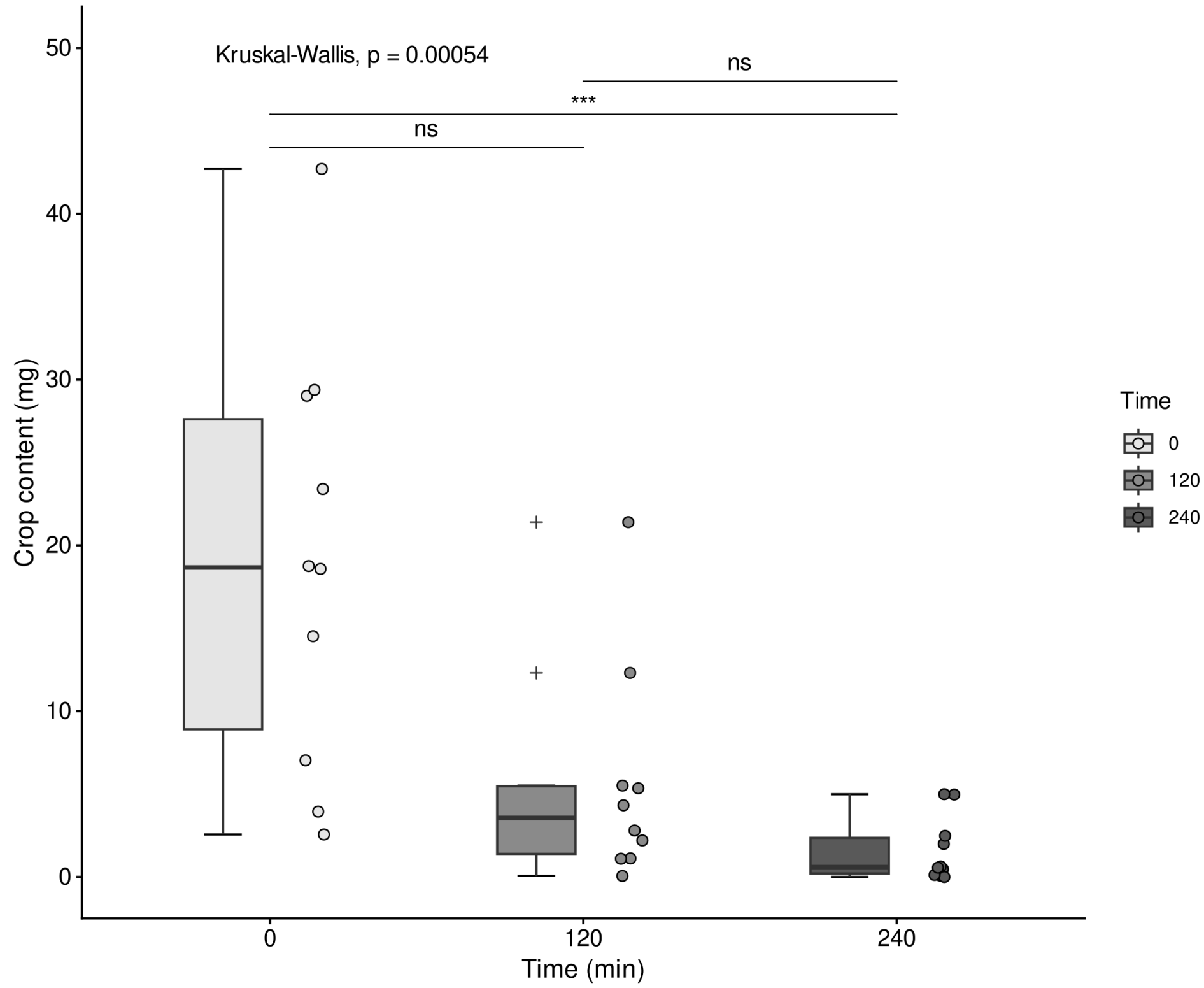

Fig. S1. To ensure the comparability of the honeybees, 30 forager bees were used to identify the time point at which their honey stomachs were empty. For this purpose, honeybees were transferred into a cage without sucrose solution. The crop content was determined as described by De˘girmenci et al.(2026) in two hour intervals using 10 bees at a time. The data did not display a normal distribution and was analyzed using the Kruskal-Wallis test ($p = 0.00054$), showing a significant difference between the time intervals. To specify these differences a Dunns post hoc analysis with Holm correction was used. Honeybees whose crop content was measured immediately after food removal (0 min) had the greatest amount of crop, with the greatest variance in crop content. After 120 min, this variance decreased. Nevertheless, the bees did not exhibit a significant decrease in crop content (Dunns test: $p_{adj} = 0.05$). It was only after 240 min that honeybees showed a significant decrease in crop content compared to those at 0 min (Dunns test: $p_{adj} = 0.0001$). It was determined that the ideal time point at which a honeybees crop stomach is empty, is four hours after the food removal. Boxplots show median ± interquartile Range (left part) and individual data points (right). Boxplots and data points are color-coded according to time intervals. Outliers are represented as crosses. $*** = p_{adj} < 0.001$, $ns = p_{adj} \geq 0.05$.

Reference

De˘girmenci, L., Gabel, M., Scheiner, R., Strehl, J. (2026). A fast and easy mass sampling method for social bee crop content. *bioRxiv*. doi=10.64898/2026.01.27.701953.

Journal of Experimental Biology • Supplementary information

Table S1. Overview of all feeding trials performed for the sucralose control experiment. For each trial the number of bees *offered* each solution and the number that *ingested* it is given, together with mortality. Because acceptance of the non-caloric sucralose was low a large number of animals had to be screened. Per trial, three bees per solution were taken into the thermogenesis assay.

	Trial	Sucrose 30%		Sucralose 1%		Died during test	Died before test
		Offered	Ingested	Offered	Ingested		
Forager	1	3	3	15	4	0	0
	2	3	3	15	5	0	4
	3	3	3	16	3	0	3
	4	3	3	18	3	0	1
	5	4	3	17	2	3	1
	6	3	3	16	4	0	3
	7	4	3	18	3	0	0
	Σ	**23**	**21**	**115**	**24**	**3**	**12**
Nurse	1	3	3	16	4	0	3
	2	3	3	18	5	0	1
	3	3	3	12	3	0	0
	4	3	3	18	3	0	0
	5	3	3	18	3	0	0
	6	3	3	13	4	1	2
	Σ	**18**	**18**	**95**	**22**	**1**	**6**

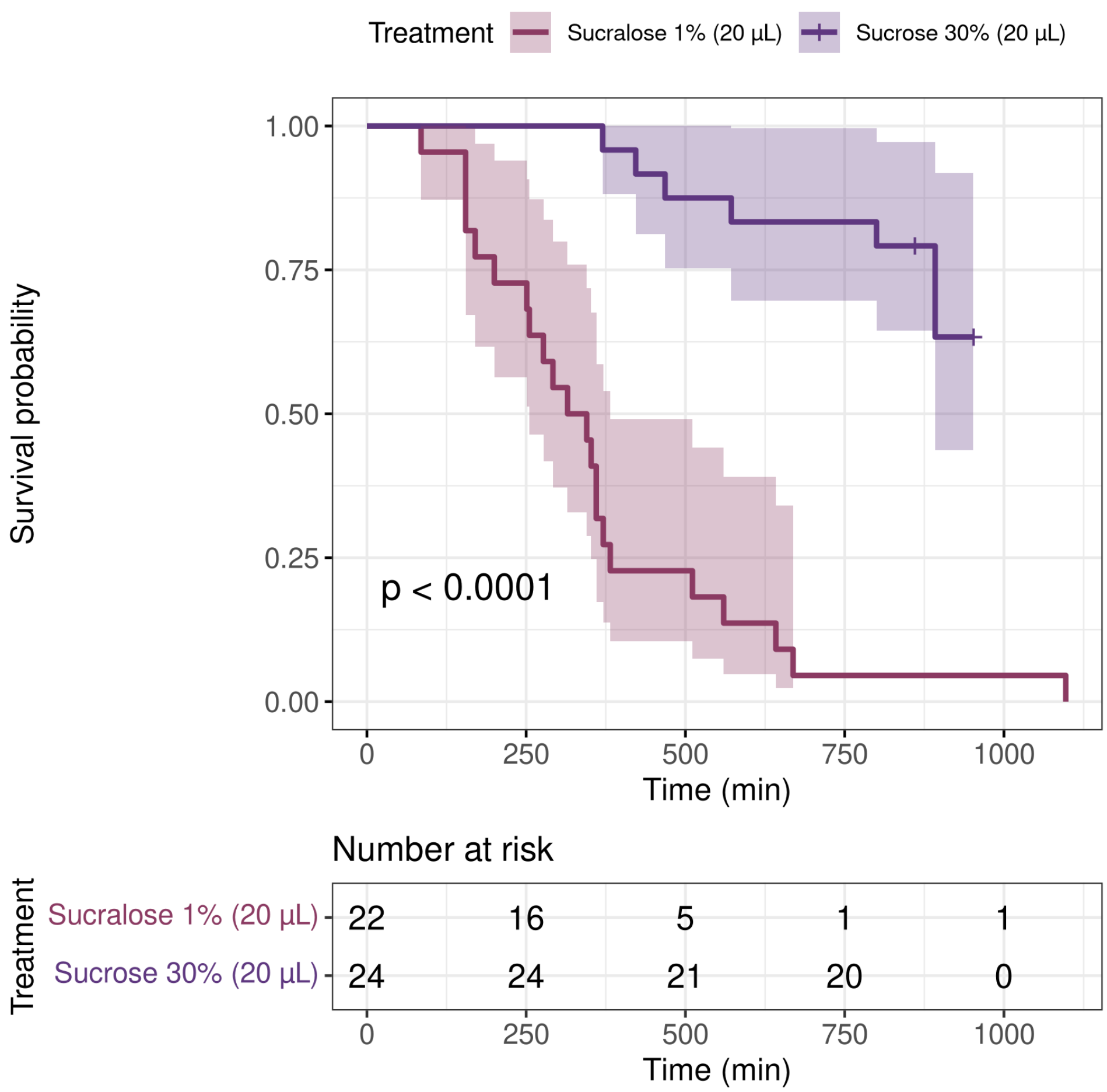

Fig. S2. To assess whether sucralose is a putative energy source for *Apis mellifera*, a survival assay was performed comparing 20 µL of 1 % sucralose (n = 22) vs 20 µL of 30 % sucrose (n = 24). Prior to feeding, honeybees were starved for 4 h and mounted in PER holders as described above. Feeding was performed in sequential batches (sucralose: 4 batches, sucrose: 2 batches) to ensure adequate sample sizes, since the acceptance of sucralose is generally low (Table S1). Survival was monitored at 15 min intervals. Data were analyzed using a Kaplan-Meier estimator and compared between treatments using a log-rank test. All sucralose-fed bees died within the observation period (median survival = 330 min, 95 % confidence interval = 255–382), whereas only 29.2 % of sucrose-fed bees (7/24) had died by the endpoint, with the remaining 17 individuals right-censored. Survival differed significantly between treatments (log-rank test: $\chi^2 = 35.4$, df = 1, $p < 0.001$).

Journal of Experimental Biology • Supplementary information
